# Association of *MICA* and *MICB* polymorphisms with the susceptibility of leukemia in Korean patients

**DOI:** 10.1038/s41408-018-0092-5

**Published:** 2018-06-12

**Authors:** In-Cheol Baek, Dong-Hwan Shin, Eun-Jeong Choi, Hyoung-Jae Kim, Jae-Ho Yoon, Byung-Sik Cho, Yoo-Jin Kim, Seok Lee, Woo-Sung Min, Hee-Je Kim, Tai-Gyu Kim

**Affiliations:** 1 Hematopoietic Stem Cell Bank, College of Medicine, The Catholic University of Korea, Seoul, Korea; 2 Department of Microbiology, College of Medicine, The Catholic University of Korea, Seoul, Korea; 3 Department of Internal Medicine, Catholic Blood and Marrow Transplantation Center, Leukemia Research Institute, College of Medicine, The Catholic University of Korea, Seoul, Korea

Hematologic malignancies including acute lymphocytic (lymphoblastic) leukemia (ALL), acute myeloid leukemia (AML), and myelodysplastic syndrome (MDS) are clonal diseases, which commonly manifest as a result of dysregulation of hematopoiesis due to genetic damage^1^. The MHC class I chain-related genes A and B (*MICA* and *MICB*), which are located on the human chromosome 6, encode proteins with 3 extracellular domains, a transmembrane segment, and a carboxy-terminal cytoplasmic tail. The extracellular domains consist of α1, α2, and α3 chains that flexibly function as a ligand for Natural killer group 2D (NKG2D), γδ T cells, and αβ CD8 T cells. *MICA* polymorphisms have been identified in over 100 alleles. Over 40 alleles have been sequenced for exons 2–6 of *MICB* (IMGT/HLA database; http://www.ebi.ac.uk/imgt/hla). The role of *MICA* and *MICB* in human disease is mainly defined by its allele associations with cancer, autoimmune disorders, and success rates of hematopoietic stem cell transplantation (HSCT)^2–5^. In this study, we analyzed the possible association of *MICA* and *MICB* polymorphisms with ALL, AML, and MDS. Our results also reveal a correlation between amino acid variants in MICA external domains and ALL, AML, and MDS.

We randomly selected a total of 324 patients diagnosed with ALL, AML, and MDS who were treated at the Department of Internal Medicine, Catholic Blood and Marrow Transplantation Center at Seoul St. Mary’s Hospital, between November 2010 and April 2013, and enrolled 324 patients with ALL, AML, and MDS (53.7% male; age [mean ± SD] 43 ± 13 years) included 85 ALL (58.8% male; age 39 ± 13 years), 172 AML (47.7% male; age 43 ± 13 years), and 67 MDS (62.7% male; age 47 ± 13 years). Diagnoses were based on the criteria specified by World Health Organization^6^. The study was performed in accordance with the Declaration of Helsinki. The patients with ALL, AML, and MDS and 200 ethnically matched non-leukemic controls signed appropriate informed consent to participate. The use of the material was reviewed and approved by the local institutional review board (IRB) of The Catholic University of Korea, with written informed consent obtained for all samples collected (IRB Number: SCMC07BR131). DNA was isolated from peripheral blood. We applied sequence-based typing (PCR-SBT) as an authentication method to amplify the exons 2–5 of *MICA*^7^. Genotyping was performed by PCR-SBT^4^ for *MICA*-129 alleles (A to G) in exon 3 at position 454 of the *MICA* gene. *MICB* primers were designed based on exons 2–4 for genotyping of *MICB*-sequence alleles^8^. The *χ*^2^ test and Fisher’s exact test were used to determine statistical significance. *P*-values were corrected (*P*_c_) using the Bonferroni’s method. The odds ratio (OR) was calculated using Haldane’s modification of Woolf’s method. Hardy-Weinberg equilibrium in controls was analyzed for each SNP using SNPStats on the website (http://bioinfo.iconcologia.net/snpstats/start.htm) (*P* > 0.05).

In total patients, the frequencies of MICA*002:01 alleles and A9 alleles were higher than in controls (112 of 324 [34.6%] versus 42 of 200 [21.0%]; *P*_c_ = 0.012, OR = 2.0, 95% confidence interval [95% CI] 1.3–3.0, 118 of 324 [36.4%] versus 44 of 200 [22.0%]; *P*_c_ = 0.003, OR = 2.0, 95% CI 1.3–2.9). However, the A5.1 (67 of 324 [21.0%] versus 76 of 200 [38.0%]; *P*_c_ < 0.001, OR = 0.4, 95% CI 0.3–0.6) showed lower frequencies than in the controls. These significant associations were also found in the ALL, AML, and MDS groups. MICA*008:01 allele showed lower frequencies (for ALL (12 of 85 [14.1%] versus 74 of 200 [37.0%]): *P*_c_ = 0.002, OR = 0.3, 95% CI 0.1–0.6; for AML (72 of 172 [41.9%] versus 74 of 200 [37.0%]): *P*_c_ = 0.042, OR = 0.5, 95% CI 0.3–0.8; for MDS (11 of 67 [16.4%] versus 74 of 200 [37.0%]): *P*_c_ = 0.026, OR = 0.3, 95% CI 0.2–0.7). The frequencies of *MICA*-STR A9 alleles were higher in patients with ALL, AML, and MDS than in the controls (for ALL (34 of 85 [40.0%] versus 44 of 200 [22.0%]): *P*_c_ = 0.007, OR = 2.4, 95% CI 1.4–4.1; for AML (60 of 172 [34.9%] versus 44 of 200 [22.0%]): *P*_c_ = 0.023, OR = 1.9, 95% CI 1.2–3.0). *MICA* A5.1 alleles were less frequent than in the controls (for ALL (13 of 85 [15.3%] versus 76 of 200 [38.0%]): *P*_c_ < 0.001, OR = 0.3, 95% CI 0.2–0.6; for AML (42 of 172 [25.0%] versus 76 of 200 [38.0%]): *P*_c_ = 0.037, OR = 0.5, 95% CI 0.3–0.8; for MDS (12 of 67 [17.9%] versus 76 of 200 [38.0%]): *P*_c_ = 0.012, OR = 0.4, 95% CI 0.2–0.7). Interestingly, a methionine (ATG) to valine (GTG) change at position 129 of the α2-heavy chain domain categorized the *MICA* alleles into strong (*MICA*-129 met) and weak (*MICA*-129 val) binders of the NKG2D receptor^5^. We found significant data for the *MICA* external domain, especially that of the protein encoded by *MICA*-129. There are non-synonymous polymorphic sites covering exons 2–4, that encode non-conservative amino acid substitutions in MICA external domains^2^. Codon 14 of MICA protein including MICA*002:01 showed strong odds ratios (1.8 ~ 2.7) and corrected *P*-value (*P*_c_ < 0.048). The frequency of the MICB*005:03 of 10 *MICB* sequence alleles was lower in patients than in the controls (for total (9 of 324 [2.8%] versus 21 of 200 [10.5%]): *P*_c_ = 0.002, OR = 0.2, 95% CI 0.1–0.5; for AML (5 of 172 [2.9%] versus 21 of 200 [10.5%]): *P*_c_ = 0.024, OR = 0.3, 95% CI 0.1–0.7) (Table 1).

**Table 1:** Frequencies of *MICA* and *MICB* genotypes, *MICA*-STR, MICA-14, and -129 amino acid alleles (*P*_c_ *<* 0.05)

	Controls	Total patients	ALL	AML	MDS
*MICA* or *MICB*	*n* = 200 (%)	*n* = 324 (%)	*n* = 85 (%)	*n* = 172 (%)	*n* = 67 (%)
*MICA* genotype					
*002:01	42 (21.0)	112 (34.6)^a^	31 (36.5)	58 (33.7)	23 (34.3)
*008:01	74 (37.0)	62 (19.4)	12 (14.1)^h^	39 (23.3)^m^	11 (16.4)^t^
*MICA*-STR alleles					
A5.1	76 (38.0)	67 (21.0)^b^	13 (15.3)^i^	42 (25.0)^n^	12 (17.9)^u^
A9	44 (22.0)	118 (36.4)^c^	34 (40.0)^j^	60 (34.9)^o^	24 (35.8)
*MICA*-14 amino acid					
Gly	49 (24.5)	129 (39.8)^d^	40 (47.1)^k^	63 (36.6)^p^	26 (38.8)^v^
*MICA*-129 amino acid					
Met	98 (49.0)	192 (59.3)^e^	58 (68.2)^l^	91 (52.9)^¥^	43 (64.2)
Val	185 (92.5)	277 (85.5)^f^	72 (84.7)	148 (86.0)	57 (85.1)
*MICB* genotype					
*005:03	21 (10.5)	9 (2.8)^g^	2 (2.4)	5 (2.9)^q§^	2 (3.0)

Controls versus total patients: ^a^OR = 2.0 (1.3–3.0), *P*_c_ = 0.014; ^b^OR = 0.4 (0.3–0.6), *P*_c_ < 0.001; ^c^OR = 2.0 (1.3–2.9), *P*_c_ = 0.003; ^d^OR = 2.0 (1.4–3.0), *P*_c_ < 0.001; ^e^OR = 1.5 (1.1–2.2), *P*_c_= 0.043; ^f^OR = 0.5 (0.3–0.9), *P*_c_ = 0.032; ^g^OR = 0.2 (0.1–0.5), *P*_c_ = 0.002

Controls versus ALL: ^h^OR = 0.3 (0.1–0.6), *P*_c_ = 0.002; ^i^OR = 0.3 (0.2–0.6), *P*_c_ < 0.001; ^j^OR = 2.4 (1.4–4.1), *P*_c_ = 0.009; ^k^OR = 2.7 (1.6–4.7), *P*_c_ < 0.001; ^l^OR = 2.2 (1.3–3.8), *P*_c_ = 0.006

Controls versus AML: ^m^OR = 0.5 (0.3–0.8), *P*_c_= 0.042; ^n^OR = 0.5 (0.3–0.8), *P*_c_= 0.037; ^o^OR = 1.9 (1.2–3.0), *P*_c_= 0.029; ^p^OR = 1.8 (1.1–2.8), *P*_c_ = 0.022; ^q^OR = 0.3 (0.1–0.7), *P*_c_= 0.024

Controls versus MDS: ^t^OR = 0.3 (0.2–0.7), *P*_c_= 0.026; ^u^OR = 0.4 (0.2–0.7), *P*_c_= 0.012; ^v^OR = 2.0 (1.1–3.5), *P*_c_= 0.048

*ALL* acute lymphocytic leukemia, *AML* acute myeloid leukemia, *MDS* myelodysplastic syndrome, *P* Pearson’s test, *P*_c_ Bonferroni’s correction, *STR* short tandem repeat

^§^Fisher’s exact test, ^¥^*MICA*-129 A allele of AML was different from that of ALL (*P* = 0.019, *P*_c_ = 0.038)

As an important ligand of human NKG2D, an activating cell surface receptor expressed on NK and T cells, the MICA protein is expressed on several tumors, especially epithelium-derived cancer cells^5^. Ligand binding of MICA to the NKG2D receptor activates the innate immune response by stimulating NK and γδT cells. Tumor cells expressing high levels of membrane MICA and other NKG2D ligands are rejected by NK and CD8αβT cells, thereby stimulating antitumor activity, while reduced expression of membrane MICA as well as increased soluble MICA in serum leads to inactivation of the NKG2D-mediated antitumor response^9^. Recently, it has been shown that soluble NKG2D ligands can activate NKG2D-dependent signaling, rather than lead to inactivation^10^. It is assumed that the reduction of membrane MICA encoded by the MICA*002:01 and A9 alleles, by release or shedding from the cell surface, potentially reduces the immunogenic signals of tumor cells, resulting in these tumor cells becoming less detectable by NK and T cells.

NK cytotoxicity experiments have shown that this downregulation-resisting allele, MICA*008:01, is functionally relevant and may aid in the elimination of virus-infected cells. MICA*008:01 and A5.1 are the most common alleles in population studies^2^. We can infer that, at least to some extent, the allelic repertoire of *MICA* represents an evolutionary record of past pathogen-driven selection and that the protective role of MICA*008:01 and A5.1 casts a ‘selective’ effect on the distribution of this allele.

Although the *MICA*-129 val/val genotype and elevated sMICA serum levels post-HSCT are independently associated with the incidence of cGVHD (*P* = 0.002 and 0.001), regardless of the history of acute GVHD, the presence of MICA Abs before transplantation confers protection against cGVHD (*P* = 0.04)^4^. There is an inverse relationship between UC or cGVHD and total patients including ALL, suggesting that these genetic characteristics of *MICA* influence disease incidence.

With the typing of MICA*002:01, MICA*008:01, and MICB*005:03 alleles associated with ALL, AML, and MDS using previously developed microarrays^11,12^, it will be possible to predict within more short time whether they are at risk or not. Furthermore, the investigation of *MIC* alleles in individual tumor samples, at the level of protein expression, should be of further interest. Our results illustrate that MICA-NKG2D played a role in disease pathogenesis in the majority of patients in our cohort of cases of ALL, AML, and MDS, and further investigation into this signaling axis may reveal potent therapeutic targets.
